# Effect of dapagliflozin on ventricular arrhythmias, resuscitated cardiac arrest, or sudden death in DAPA-HF

**DOI:** 10.1093/eurheartj/ehab560

**Published:** 2021-08-27

**Authors:** James P Curtain, Kieran F Docherty, Pardeep S Jhund, Mark C Petrie, Silvio E Inzucchi, Lars Køber, Mikhail N Kosiborod, Felipe A Martinez, Piotr Ponikowski, Marc S Sabatine, Olof Bengtsson, Anna Maria Langkilde, Mikaela Sjöstrand, Scott D Solomon, John J V McMurray

**Affiliations:** British Heart Foundation Cardiovascular Research Centre, University of Glasgow, Scotland, UK; British Heart Foundation Cardiovascular Research Centre, University of Glasgow, Scotland, UK; British Heart Foundation Cardiovascular Research Centre, University of Glasgow, Scotland, UK; British Heart Foundation Cardiovascular Research Centre, University of Glasgow, Scotland, UK; Section of Endocrinology, Yale School of Medicine, New Haven, CT, USA; Department of Cardiology, Rigshospitalet, Copenhagen University Hospital, Copenhagen, Denmark; Saint Luke’s Mid America Heart Institute, University of Missouri, Kansas City, MO, USA; The George Institute for Global Health, University of New South Wales, Sydney, Australia; Universidad Nacional de Córdoba, Córdoba, Argentina; Center for Heart Diseases, University Hospital, Wroclaw Medical University, Wroclaw, Poland; TIMI Study Group, Brigham and Women's Hospital, Boston, MA, USA; D ivision of Cardiovascular Medicine, Brigham and Women’s Hospital, Boston, MA, USA; Lat e Stage Development, Cardiovascular, Renal and Metabolism, BioPharmaceuticals R&D, AstraZeneca, Gothenburg, Sweden; Lat e Stage Development, Cardiovascular, Renal and Metabolism, BioPharmaceuticals R&D, AstraZeneca, Gothenburg, Sweden; Lat e Stage Development, Cardiovascular, Renal and Metabolism, BioPharmaceuticals R&D, AstraZeneca, Gothenburg, Sweden; D ivision of Cardiovascular Medicine, Brigham and Women’s Hospital, Boston, MA, USA; British Heart Foundation Cardiovascular Research Centre, University of Glasgow, Scotland, UK

**Keywords:** Sodium-glucose cotransporter 2 inhibitor, Heart failure, Ventricular tachyarrhythmia, Sudden death

## Abstract

**Aims:**

The aim of this study was to examine the effect of dapagliflozin on the incidence of ventricular arrhythmias and sudden death in patients with heart failure and reduced ejection fraction (HFrEF).

**Methods and results:**

In a *post hoc* analysis of DAPA-HF, we examined serious adverse event reports related to ventricular arrhythmias or cardiac arrest, in addition to adjudicated sudden death. The effect of dapagliflozin, compared with placebo, on the composite of the first occurrence of any serious ventricular arrhythmia, resuscitated cardiac arrest, or sudden death was examined using Cox proportional hazards models. A serious ventricular arrhythmia was reported in 115 (2.4%) of the 4744 patients in DAPA-HF (ventricular fibrillation in 15 patients, ventricular tachycardia in 86, ‘other’ ventricular arrhythmia/tachyarrhythmia in 12, and torsade de pointes in 2 patients). A total of 206 (41%) of the 500 cardiovascular deaths occurred suddenly. Eight patients survived resuscitation from cardiac arrest. Independent predictors of the composite outcome (first occurrence of any serious ventricular arrhythmia, resuscitated cardiac arrest or sudden death), ranked by chi-square value, were log-transformed N-terminal pro-B-type natriuretic peptide, history of ventricular arrhythmia, left ventricular ejection fraction, systolic blood pressure, history of myocardial infarction, male sex, body mass index, serum sodium concentration, non-white race, treatment with dapagliflozin, and cardiac resynchronization therapy. Of participants assigned to dapagliflozin, 140/2373 patients (5.9%) experienced the composite outcome compared with 175/2371 patients (7.4%) in the placebo group [hazard ratio 0.79 (95% confidence interval 0.63–0.99), *P* = 0.037], and the effect was consistent across each of the components of the composite outcome.

**Conclusions:**

Dapagliflozin reduced the risk of any serious ventricular arrhythmia, cardiac arrest, or sudden death when added to conventional therapy in patients with HFrEF.

**Clinical trial registration:**

ClinicalTrials.gov unique identifier: NCT03036124 (DAPA-HF).

## Graphical Abstract

**Figure:**
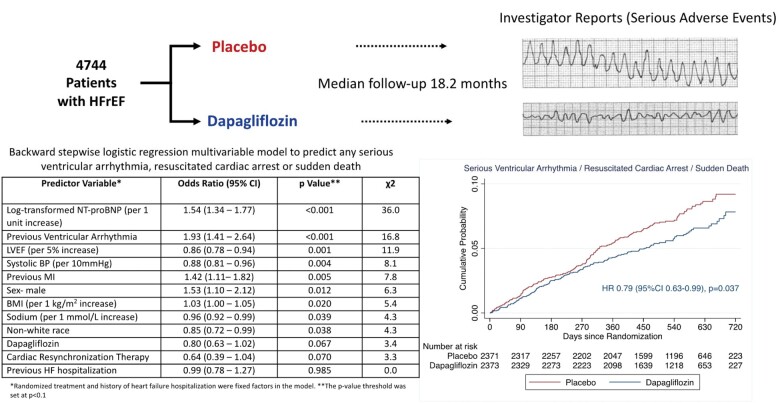
DAPA-HF: trial design and main findings. BMI, body mass index; BP, blood pressure; CI, confidence interval; HF, heart failure; HFrEF, heart failure and reduced ejection fraction; HR, hazard ratio; LVEF, left ventricular ejection fraction; MI, myocardial infarction; NT-proBNP, N-terminal pro-B-type natriuretic peptide.

**See page 3739 for the editorial comment on this article (doi:10.1093/eurheartj/ehab563)**

## Introduction

Sodium-glucose cotransporter 2 (SGLT2) inhibitors have recently been shown to reduce both worsening heart failure and death from cardiovascular causes in patients with heart failure and reduced ejection fraction (HFrEF).^1–4^ Ventricular arrhythmias are common and are one of the key causes of death in HFrEF, as indicated by the benefit of implantable cardioverter defibrillators (ICDs) on sudden death.^5–9^ Although rates of sudden death have been declining over the past three decades with improving pharmacological therapy, this mode of death remains the principal cause of mortality in ambulatory patients with HFrEF, particularly those with mild symptoms.^10^

It is important, therefore, to investigate the effect of new therapies for HFrEF on ventricular arrhythmias and mode of death, including sudden death. Various proposed actions of SGLT2 inhibitors raise the hypothesis that these agents might reduce the risk of ventricular arrhythmias. Potential antiarrhythmic actions include favourable effects on left ventricular loading conditions and remodelling, autonomic nervous system activity, serum electrolytes, the cardiac sodium channel current (late /_Na_), and the myocardial sodium-hydrogen (Na^+^/H^+^) exchanger.^11–19^

Therefore, to investigate the hypothesis that SGLT2 inhibition reduces the incidence of ventricular arrhythmias, we undertook a *post hoc* analysis of DAPA-HF, examining serious adverse event (SAE) reports of ventricular arrhythmias, resuscitated cardiac arrest, and sudden death according to randomized assignment to dapagliflozin or placebo.

## Methods

### Study design and participants

DAPA-HF was a randomized, double-blind, controlled trial in patients with HFrEF, which evaluated the efficacy and safety of dapagliflozin 10 mg once daily, compared with matching placebo, added to standard care. The design, baseline characteristics, and primary results of the trial are published.^1^ ^,^ ^20^ ^,^ ^21^ Ethics Committees at each of the 410 participating institutions (in 20 countries) approved the protocol, and all patients gave written informed consent.

#### Study patients

Men and women aged ≥18 years with heart failure were eligible if they were in New York Heart Association (NYHA) functional class II–IV, had a left ventricular ejection fraction (LVEF) ≤40%, and were optimally treated with pharmacological and device therapy for HFrEF. Participants were also required to have an elevated N-terminal pro-B-type natriuretic peptide (NT-proBNP) concentration. Key exclusion criteria included: symptoms of hypotension or systolic blood pressure <95 mmHg, estimated glomerular filtration rate (eGFR) <30 mL/min/1.73 m^2^ (or rapidly declining renal function), type 1 diabetes mellitus, and another condition likely to prevent patient participation in the trial or greatly limit life expectancy. A full list of exclusion criteria is provided in the design paper.

#### Study procedures

After the provision of informed consent, visit 1 started a 14-day screening period during which the trial inclusion and exclusion criteria were checked, and baseline information was collected. Visit 2 was the randomization visit and after randomization, follow-up visits took place at 14 and 60 days, and then at 120, 240, and 360 days and every 4 months thereafter.

### Prespecified trial outcomes

The primary outcome in DAPA-HF was the composite of an episode of worsening heart failure or cardiovascular death, whichever occurred first. Secondary endpoints included the composite of the occurrence of heart failure hospitalization or cardiovascular death and all-cause death. The primary composite outcome in the present analysis was the time-to-first occurrence of any ‘serious’ ventricular arrhythmia, resuscitated cardiac arrest, or sudden death, as discussed in more detail below.

### Adjudication of death

All deaths were adjudicated according to prespecified criteria and subclassified as due to a cardiovascular cause, a non-cardiovascular cause, or an undetermined cause. Cardiovascular deaths were further subclassified into the following categories: sudden death, heart failure or cardiogenic shock (‘pump failure’), acute myocardial infarction, stroke, and others (e.g. procedure-related). Sudden death included: (i) witnessed and instantaneous death without new or worsening symptoms, (ii) death witnessed within 60 min of the onset of new or worsening cardiac symptoms, (iii) death witnessed and attributed to an identified arrhythmia, e.g. captured on an electrocardiographic recording or witnessed on a monitor, or unwitnessed but found on ICD review, (iv) death after unsuccessful resuscitation from cardiac arrest or a patient successfully resuscitated from cardiac arrest but who died within 24 h without identification of a non-cardiac aetiology, (v) death >1 day after a patient has been successfully resuscitated from cardiac arrest and without identification of a non-cardiovascular aetiology, and (vi) unwitnessed death in a subject seen alive and clinically stable ≤24 h before being found dead without any evidence supporting a specific non-cardiovascular cause of death.

### Identification of ventricular arrhythmias reported as serious adverse events

Because of the extensive safety information on dapagliflozin and SGLT2 inhibitors, selective adverse event reporting was employed in DAPA-HF. While all SAEs were collected, only non-SAEs associated with discontinuation of trial treatment and prespecified ‘non-SAEs of interest’ (specifically, volume depletion, renal events, major hypoglycaemic events, bone fractures, diabetic ketoacidosis, and amputations) were collected.

Serious adverse events were examined for any report of any ventricular arrhythmia. The adverse events were identified using the Medical Dictionary for Regulatory Activities (MedDRA) preferred terms ‘ventricular tachycardia’ (VT) (including sustained and non-sustained VT), ‘ventricular fibrillation’ (VF), ‘torsade de pointes’, ‘ventricular tachyarrhythmia’, and ‘ventricular arrhythmia’.

### Analysis of ventricular arrhythmias and sudden death

The primary outcome in the present analysis was the time-to-first occurrence of any ‘serious’ ventricular arrhythmia, resuscitated cardiac arrest, or sudden death. For this analysis, a ‘serious’ ventricular arrhythmia was defined as VT, VF, torsade de pointes, ventricular tachyarrhythmia and ventricular arrhythmia (reflecting MedDRA preferred terms used for reporting SAEs). Premature ventricular ectopic beats were not included in this analysis. For participants who experienced more than one type of ventricular arrhythmia, only the first event reported was included in the analysis of the composite endpoint.

Serious adverse event reports of cardiac arrest were also examined and patients who were resuscitated from cardiac arrest were included in this analysis if the cardiac arrest was adjudicated as being non-fatal.

### Statistical analysis

Baseline characteristics were compared for patients experiencing any component of the composite outcome of any serious ventricular arrhythmia, resuscitated cardiac arrest, or sudden death and patients who did not. Categorical variables are reported as whole numbers with percentages. Continuous variables are reported by their mean value with standard deviations or median value plus interquartile ranges depending on a respective normal or skewed distribution. The effect of dapagliflozin compared with placebo was examined in a time-to-first event analysis using Cox proportional hazards regression models. The primary models included factors for randomized treatment group assignment and history of heart failure hospitalization and the randomization stratification variable of diabetes status. In addition, we examined the effect of dapagliflozin, compared with placebo, on the narrower composite of VT, VF, or torsade de pointes (i.e. excluding the MedDRA preferred terms ‘ventricular tachyarrhythmia’ and ‘ventricular arrhythmia’). In sensitivity analyses, we excluded non-sustained VT from both the primary composite outcome and the narrower composite. Event rates per 100 person-years were calculated and are presented with 95% confidence intervals (CIs). The cumulative incidences of outcomes are presented graphically using the Kaplan–Meier method. The effect of randomized treatment was examined in Cox proportional hazards regression models, and the interaction with randomized therapy tested in several subgroups: patients with an ischaemic or non-ischaemic aetiology for heart failure, patients with or without an implanted defibrillating device [ICD or cardiac resynchronization therapy with a defibrillator (CRT-D)] at baseline, patients treated with or not treated with a mineralocorticoid receptor antagonist (MRA), patients with or without diabetes, patients with or without a history of ventricular arrhythmia, patients with a diagnosis of heart failure within 1 year or for >1 year before randomization, and patients who were in NYHA class II or III/IV. Baseline LVEF, NT-proBNP, and systolic blood pressure subgroups were analysed both as a categorical variable (divided at the median) and a continuous variable (using fractional polynomial models). These variables were chosen because: (i) patients with an ischaemic aetiology are at greater risk of sudden death than those with a non-ischaemic aetiology and there seems to be an interaction between aetiology and the effectiveness of an ICD. (ii) We wanted to examine the effectiveness of dapagliflozin added to other treatments reducing the risk of ventricular arrhythmias/sudden death. The only pharmacological treatment subgroup reasonable to do this was MRA therapy (∼70% yes/30% no); the other relevant therapies had too many (≥94% on a renin-angiotensin system blocker and a beta-blocker) or too few receiving the treatment [angiotensin receptor-neprilysin inhibitor (ARNI) 11%]. (iii) The ICD/CRT-D subgroup was chosen for the same reason as (ii) and we wondered if dapagliflozin might have more effect in patients without an ICD/CRT-D. (iv) NYHA class, NT-proBNP, systolic blood pressure, and time from diagnosis of heart failure were all chosen as recognized markers of severity of heart failure; ICDs are not indicated in patients with more severe heart failure symptoms because of the relatively higher competing risk of pump-failure death and we were interested to see whether dapagliflozin was less effective in reducing ventricular arrhythmias/sudden death in patients with evidence of more severe/advanced heart failure. (v) LVEF is a well-known predictor of sudden death and arrhythmias and the primary determinant of whether there is an indication for an ICD. (vi) Patients with a previous ventricular arrhythmia were a subset at high risk of the events of interest. (vii) Diabetes mellitus—this was included because HFrEF patients with diabetes have a higher risk of sudden death than those without diabetes and ICDs seem to be less effective in patients with diabetes. To account for the fact that death precludes the future occurrence of ventricular arrhythmias, a proportional hazards competing-risk regression model was used as a sensitivity analysis.^22^ To examine the relative hazard of clinical outcomes before or after the occurrence of any serious ventricular arrhythmia, Cox proportional hazards regression models were performed with the occurrence of the serious ventricular arrhythmia outcome modelled as a time-varying covariate.^23^ A multivariable model was adjusted for factors known to influence prognosis including beta-blocker use, angiotensin-converting enzyme inhibitor or angiotensin receptor blocker use, ARNI use, MRA use, ischaemic aetiology, LVEF, presence of an ICD or a CRT device, NYHA class, hypertension, diabetes, history of hospitalization for heart failure, log-transformed NT-proBNP, and atrial fibrillation.

The association between relative change in NT-proBNP from baseline to 8 months (using log_2_-transformed values), adjusted for baseline NT-proBNP, and the incidence of serious ventricular arrhythmia, resuscitated cardiac arrest, or sudden death was examined using Cox proportional hazards linear models. For this analysis, only events occurring after 8 months were included. The association between baseline LVEF and NT-proBNP as continuous variables and the primary composite outcome were examined in linear models and reported graphically.

A backward stepwise logistic regression multivariable model was constructed with a *P*-value threshold of <0.1 to identify baseline variables predicting the occurrence of the composite serious ventricular arrhythmia, resuscitated cardiac arrest, or sudden death. Variables included in the model were: age, region, sex, QRS duration, eGFR, race, systolic blood pressure, heart rate, body mass index, LVEF, log-transformed NT-proBNP, serum potassium, serum sodium, NYHA functional class, ischaemic aetiology for heart failure, Kansas City Cardiomyopathy Questionnaire (KCCQ) total symptom score, history of ventricular arrhythmia, hypertension, diabetes, atrial fibrillation, chronic obstructive pulmonary disease, myocardial infarction, stroke, percutaneous coronary intervention or coronary bypass grafting, ICD, CRT, and anaemia. Randomized treatment and prior heart failure hospitalization were fixed factors in the model. Odds ratios with 95% CIs are presented. The effect of individual variables on the logistic regression model was examined and a *χ* ^2^ value calculated using a post-estimation test. Randomization treatment and a history of heart failure hospitalization were forced into the model.

A *P*-value of <0.05 was considered statistically significant. Statistical analyses were performed using Stata 17 (Stata Corp., College Station, TX, USA).

## Results

At least one episode of a serious ventricular arrhythmia was reported in 115 (2.4%) of the 4744 patients in DAPA-HF. The events reported were VT in 87 patients (86 as a first event), VF in 16 patients (15 as a first event), torsade de pointes in 2 patients (2 as a first event), a ‘ventricular tachyarrhythmia’ in 1 patient (1 as a first event), and a ‘ventricular arrhythmia’ in 13 patients (11 as a first event). Among patients experiencing VT, 7 patients reported to have a ‘non-sustained VT’ event (6 as a first event) and 4 reported a ‘paroxysmal VT’ event (3 as a first event), identified by MedDRA low-level terms. Of the 500 cardiovascular deaths, 206 (41%) were adjudicated as sudden death. Overall, 23 patients (0.5%) had a documented cardiac arrest, of whom 8 (35%) survived. A total of 315 patients (6.6%) experienced the composite of a serious ventricular arrhythmia (104 as the first event in the composite), resuscitated cardiac arrest (8 as the first event), or sudden death (203 as the first event). A total of 8 patients had the same time to the occurrence of a serious ventricular arrhythmia and sudden death and were counted as sudden death in the analysis of the composite outcome. Similarly, one patient who had the same time to the occurrence of a serious ventricular arrhythmia and resuscitated cardiac arrest was counted as a resuscitated cardiac arrest in the analysis of the primary composite outcome.

### Baseline characteristics

*Table 1* shows the baseline characteristics of patients who did and did not experience the primary composite outcome in this analysis (serious ventricular arrhythmia, resuscitated cardiac arrest, or sudden death). Compared to patients who did not experience the composite outcome, patients who did were more likely to be male, have a history of ischaemic heart disease and ventricular arrhythmia, and had a lower LVEF, higher NT-proBNP, longer duration of heart failure, worse renal function, a broader QRS, and greater symptom burden at baseline (as reflected by NYHA functional class and KCCQ Total Symptom Score). More patients who experienced a composite event were treated with an MRA and had an ICD at baseline. Background treatment with beta-blockers was high in both groups, although slightly fewer patients who experienced the composite outcome were taking a beta-blocker at baseline. The baseline characteristics of patients with any serious ventricular arrhythmia or sudden death, separately, are shown in Supplementary material online, *Appendix Table S1*.

**Table 1:** Baseline characteristics of participants who had no ventricular arrhythmia compared with those who had a composite of a serious ventricular arrhythmia,^a^ resuscitated cardiac arrest, or sudden death

	No serious ventricular arrhythmia, resuscitated cardiac arrest, or sudden death	Serious ventricular arrhythmia, resuscitated cardiac arrest, or sudden death	*P*-value
Number of patients	4429 (93.4)	315 (6.6)	
Age (years)	66 ± 11	66 ± 11	0.910
Race			0.065
White	3097 (69.9)	236 (74.9)	
Black	210 (4.7)	16 (5.1)	
Asian	1060 (23.9)	56 (17.8)	
Other	62 (1.4)	7 (2.2)	
Region			0.028
North America	639 (14.4)	38 (12.1)	
South America	751 (17.0)	66 (21.0)	
Europe	1999 (45.1)	155 (49.2)	
Asia-Pacific	1040 (23.5)	56 (17.8)	
Male sex	3371 (76.1)	264 (83.8)	0.002
SBP (mmHg)	122 ± 16	118 ± 15	<0.001
Heart rate (b.p.m.)—sinus rhythm	71 ± 11	71 ± 11	0.670
Heart rate (b.p.m.)—AF	73 ± 13	72 ± 12	0.410
BMI (kg/m^2^)	28 ± 6	28 ± 6	0.430
eGFR (mL/min/1.73 m^2^)	66 ± 19	63 ± 18	0.004
eGFR <60 mL/min/1.73 m^2^	1780 (40.2)	146 (46.3)	0.032
LVEF (%)	32 (26–37)	30 (24–35)	<0.001
LVEF			<0.001
≤Median	2269 (51.2)	200 (63.5)	
>Median	2160 (48.8)	115 (36.5)	
NT-proBNP (pg/mL)—not AF/F	1238 (735–2259)	2066 (995–3872)	<0.001
NT-proBNP (pg/mL)—AF/F	1777 (1099–2998)	2134 (1226–4097)	0.008
Potassium (mmol/L)	4.5 ± 0.5	4.5 ± 0.5	0.190
Sodium (mmol/L)	140 ± 3	139 ± 3	0.002
QRS duration (ms)	121 ± 36	126 ± 32	0.016
QRS duration ≥130 ms	1508 (34.0)	129 (41.0)	0.013
QRS duration ≥150 ms	964 (21.8)	81 (25.7)	0.100
NYHA class			0.002
II	3013 (68.0)	190 (60.3)	
III	1380 (31.2)	118 (37.5)	
IV	36 (0.8)	7 (2.2)	
KCCQ-TSS	78 (59–92)	72 (56–88)	0.002
Duration of heart failure			<0.001
<1 year	1049 (23.7)	49 (15.6)	
1–5 years	1681 (38.0)	110 (34.9)	
>5 years	1699 (38.4)	156 (49.5)	
Ischaemic aetiology	2477 (55.9)	197 (62.5)	0.022
Medical history			
Previous ventricular arrhythmia	460 (10.4)	60 (19.0)	<0.001
Hypertension	3291 (74.3)	232 (73.7)	0.800
Diabetes mellitus	1845 (41.7)	138 (43.8)	0.450
AF history	1693 (38.2)	125 (39.7)	0.610
AF/F on baseline ECG	1050 (23.7)	78 (24.8)	0.670
Prior HF hospitalization	2100 (47.4)	151 (47.9)	0.860
MI	1926 (43.5)	166 (52.7)	0.001
PCI	1498 (33.8)	126 (40.0)	0.026
CABG	736 (16.6)	63 (20.0)	0.120
Stroke	426 (9.6)	40 (12.7)	0.076
COPD	544 (12.3)	41 (13.0)	0.700
Anaemia^b^	1208 (27.3)	94 (29.8)	0.320
CV therapy (%)			
Loop diuretic	3549 (80.1)	276 (87.6)	0.001
Thiazide diuretic	443 (10.0)	31 (9.8)	0.930
ARNI	465 (10.5)	43 (13.7)	0.080
ACE inhibitor	2489 (56.2)	172 (54.6)	0.580
ARB	1229 (27.7)	78 (24.8)	0.250
Beta-blocker	4263 (96.3)	295 (93.7)	0.022
MRA	3128 (70.6)	242 (76.8)	0.019
Digoxin	827 (18.7)	60 (19.0)	0.870
Amiodarone	135 (3.0)	15 (4.8)	0.093
Sotalol	9 (0.2)	2 (0.6)	0.120
ICD	1140 (25.7)	102 (32.4)	0.010
CRT-D	282 (6.4)	19 (6.0)	0.810
Diabetes treatments^c^			
Biguanide	953 (51.7)	63 (45.7)	0.530
Sulphonylurea	411 (22.3)	27 (19.6)	0.670
DPP4 inhibitor	284 (15.4)	26 (18.8)	0.200
GLP-1 agonist	20 (1.1)	1 (0.7)	0.730
Insulin	503 (27.3)	37 (26.8)	0.830

Values are given as *n* (%), mean ± standard deviation, or median (interquartile range).

ACE, angiotensin-converting enzyme; AF/F, atrial fibrillation/atrial flutter; ARB, angiotensin receptor blocker; ARNI, angiotensin receptor-neprilysin inhibitor; BMI, body mass index; CABG, coronary bypass graft; COPD, chronic obstructive pulmonary disease; CRT-D, cardiac resynchronization therapy with defibrillator; CV, cardiovascular; DPP4, dipeptidyl peptidase-4; ECG, electrocardiogram; eGFR, estimated glomerular filtration rate; GLP-1, glucagon-like peptide 1; HF, heart failure; ICD, implantable cardioverter defibrillator; KCCQ-TSS, Kansas City Cardiomyopathy Questionnaire total symptom score; LVEF, left ventricular ejection fraction; MedDRA, Medical Dictionary for Regulatory Activities; MI, myocardial infarction; MRA, mineralocorticoid receptor antagonist; NT-proBNP, N-terminal pro-B-type natriuretic peptide; NYHA, New York Heart Association; PCI, percutaneous coronary intervention; SBP, systolic blood pressure.

a Serious ventricular arrhythmia was defined as any serious adverse event report using the MedDRA preferred terms ‘ventricular tachycardia’, ‘ventricular fibrillation’, ‘torsade de pointes’, ‘ventricular tachyarrhythmia’, and ‘ventricular arrhythmia’. Premature ventricular ectopic beats were excluded.

b Anaemia was defined as haemoglobin <130 g/L in males and <120 g/L in females.

c Percent is of patients with a history of diabetes.

### Predictors of any serious ventricular arrhythmia, resuscitated cardiac arrest, or sudden death

Independent predictors of the composite outcome, ranked by *χ* ^2^ value, were log-transformed NT-proBNP, history of ventricular arrhythmia, LVEF, systolic blood pressure, history of myocardial infarction, male sex, body mass index, serum sodium concentration, non-white race, treatment with dapagliflozin, and CRT (*Table 2*).

**Table 2:** Backward stepwise logistic regression multivariable model to predict any serious ventricular arrhythmia, resuscitated cardiac arrest, or sudden death

Predictor variable^a^	Odds ratio (95% CI)	*P*-value^b^	*χ* ^2^
Log-transformed NT-proBNP (per 1 unit increase)	1.54 (1.34–1.77)	<0.001	36.0
Previous ventricular arrhythmia	1.93 (1.41–2.64)	<0.001	16.8
LVEF (per 5% increase)	0.86 (0.78–0.94)	0.001	11.9
Systolic BP (per 10 mmHg increase)	0.88 (0.81–0.96)	0.004	8.1
Previous MI	1.42 (1.11–1.82)	0.005	7.8
Male sex	1.53 (1.10–2.12)	0.012	6.3
BMI (per 1 kg/m^2^ increase)	1.03 (1.00–1.05)	0.020	5.4
Sodium (per 1 mmol/L increase)	0.96 (0.92–0.99)	0.039	4.3
Non-white race	0.85 (0.72–0.99)	0.038	4.3
Dapagliflozin	0.80 (0.63–1.02)	0.067	3.4
Cardiac resynchronization therapy	0.64 (0.39–1.04)	0.070	3.3
Previous HF hospitalization	0.99 (0.78–1.27)	0.985	0.0

BMI, body mass index; BP, blood pressure; CI, confidence interval; HF heart failure; LVEF, left ventricular ejection fraction; MI, myocardial infarction; NT-proBNP, N-terminal pro-B-type natriuretic peptide.

a Variables included in the model were age, region, sex, QRS duration, estimated glomerular filtration rate, race, systolic BP, heart rate, BMI, LVEF, log-transformed NT-proBNP, serum potassium, serum sodium, New York Heart Association functional class, ischaemic aetiology for HF, Kansas City Cardiomyopathy Questionnaire total symptom score, history of ventricular arrhythmia, hypertension, diabetes, atrial fibrillation, chronic obstructive pulmonary disease, MI, stroke, percutaneous coronary intervention or coronary artery bypass grafting, implantable cardioverter defibrillator, cardiac resynchronization therapy, and anaemia. Randomized treatment and prior HF hospitalization were fixed factors in the model.

b The *P*-value threshold was set at <0.1.

When modelled continuously (using an NT-proBNP value of 1440 pg/mL, approximating to the median, as the referent value), higher NT-proBNP was associated with a greater risk of a primary composite event and baseline values below the median were associated with a lower risk. An inverse relationship was observed between baseline LVEF, modelled continuously, and the primary composite outcome (Supplementary material online, *Appendix Figures S1 and S2*).

### Effect of randomized treatment on the incidence of any serious ventricular arrhythmia, resuscitated cardiac arrest, or sudden death

**Figure 1:**
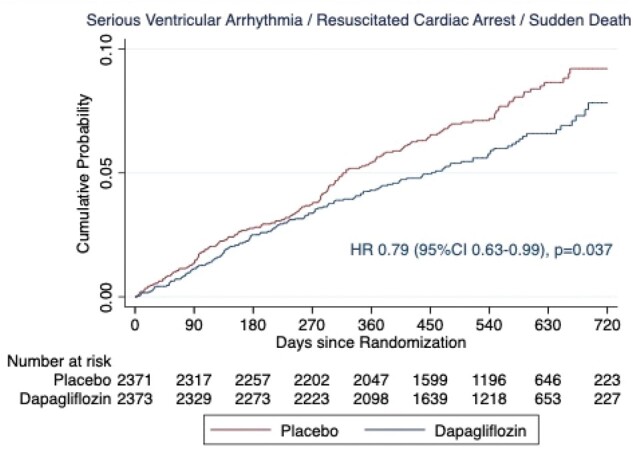
Kaplan–Meier curves for time-to-first serious ventricular arrhythmia, resuscitated cardiac arrest, or sudden death according to treatment assignment. CI, confidence interval; HR, hazard ratio.

*Table 3* shows the incidence of the composite outcome and its components, according to randomized treatment. Compared to patients randomly assigned to placebo, participants assigned to dapagliflozin had a lower rate of the composite of any serious ventricular arrhythmia, resuscitated cardiac arrest, or sudden death, whichever occurred first [hazard ratio (HR) 0.79 (95% CI 0.63–0.99), *P* = 0.037] (*Figure 1*). We conducted a sensitivity analysis including only VT, VF, and torsade de pointes as the ventricular arrhythmia component of the composite (i.e. excluding events reported as ‘ventricular tachyarrhythmia’ and ‘ventricular arrhythmia’). This analysis of VT, VF, torsade de pointes, resuscitated cardiac arrest, or sudden death also occurred less frequently in patients treated with dapagliflozin compared to placebo [HR 0.77 (95% CI 0.62–0.97), *P* = 0.025]. The effect of dapagliflozin compared to placebo on the incidence of any ‘serious’ ventricular arrhythmia [HR 0.76 (95% CI 0.53–1.10)]; VT, VF, or torsade de pointes [HR 0.72 (95% CI 0.49–1.07)]; and sudden death [HR 0.81 (95% CI 0.62–1.07)] was consistent with the effect on the primary composite outcome. In further sensitivity analyses, we excluded non-sustained VT from both the primary composite outcome and the narrower composite of VT, VF, torsade de pointes, resuscitated cardiac arrest, or sudden death; the findings of these analyses were consistent with the analyses reported above (*Table 3*). Although there were numerically more resuscitated cardiac arrests in the dapagliflozin group (5 vs. 3 in the placebo group), the overall number of events was small. Analysis modelling all-cause mortality as a competing risk also gave similar results (Supplementary material online, *Appendix Table S2*  *and*  *Figure S3*) for the primary composite outcome, the sensitivity analysis, and the analysis of the individual components of these composites.

**Table 3:** Cox proportional hazards models of clinical outcomes according to the individual components and the composite of serious ventricular arrhythmia, resuscitated cardiac arrest, or sudden death and randomized treatment

	Dapagliflozin		Placebo		
Outcome	*n/N* (%)	Event rate per 100 person-years	*n*/*N* (%)	Event rate per 100 person-years	Hazard ratio^a^ (95% CI)
Serious ventricular arrhythmia, resuscitated cardiac arrest, or sudden death	140/2373 (5.9)	4.1 (3.4–4.8)	175/2371 (7.4)	5.1 (4.4–6.0)	0.79 (0.63–0.99) *P* = 0.037
Serious ventricular arrhythmia	50/2373 (2.1)	1.4 (1.1–1.9)	65/2371 (2.7)	1.9 (1.5–2.4)	0.76 (0.53–1.10)
Resuscitated cardiac arrest	5/2373 (0.2)	0.14 (0.06–0.34)	3/2371 (0.1)	0.09 (0.03–0.27)	—
Sudden death	93/2373 (3.9)	2.7 (2.2–3.3)	113/2371 (4.8)	3.3 (2.7–3.9)	0.81 (0.62–1.07)
VT/VF/torsade de pointes/resuscitated cardiac arrest/sudden death	134/2373 (5.6)	3.9 (3.3–4.6)	171/2371 (7.2)	5.0 (4.3–5.8)	0.77 (0.62–0.97) *P* = 0.025
VT/VF/torsade de pointes	60/2373 (2.5)	1.8 (1.4–2.3)	44/2371 (1.9)	1.3 (0.9–1.7)	0.72 (0.49–1.07)
Serious ventricular arrhythmia (*minus NSVT*)/resuscitated cardiac arrest/sudden death	138/2373 (5.8)	4.0 (3.4–4.7)	169/2373 (7.1)	5.0 (4.3–5.8)	0.81 (0.64–1.01) *P* = 0.060
VT (*minus* *NSVT*)/VF/torsade de pointes/resuscitated cardiac arrest/sudden death	132/2373 (5.6)	3.8 (3.2–4.5)	165/2371 (7.0)	4.8 (4.2–5.6)	0.79 (0.63–0.99) *P* = 0.043

CI, confidence interval; NSVT, non-sustained ventricular tachycardia; VF, ventricular fibrillation; VT, ventricular tachycardia.

a Models included factors for randomized treatment, history of heart failure hospitalization and were stratified by diabetes status. A hazard ratio was not calculated where there were fewer than 10 events overall.

### Effect of dapagliflozin on any serious ventricular arrhythmia, resuscitated cardiac arrest, or sudden death in key subgroups

The effect of dapagliflozin on the composite outcome in key subgroups is shown in *Table 4*. While most showed no suggestion of heterogeneity of treatment effect, two are worthy of comment. Of the 1242 patients with a defibrillating device (ICD or CRT-D) implanted at baseline, 102 (8.2%) experienced at least one ventricular arrhythmia. Among the 3502 participants without a defibrillating device, 213 (6.1%) experienced either a serious ventricular arrhythmia, resuscitated cardiac arrest, or sudden death. The HR for the effect of dapagliflozin, compared with placebo, on this composite outcome in patients with an ICD/CRT-D was 0.99 (95% CI 0.67–1.45), compared with 0.71 (95% CI 0.54–0.93) in those without such a device. Despite the apparently smaller effect in patients with a defibrillating device, the *P*-value for interaction was not significant (=0.174).

**Table 4:** Cox proportional hazards models for a serious ventricular arrhythmia, resuscitated cardiac arrest, or sudden death according to randomized treatment assignment in key patient subgroups

	Dapagliflozin		Placebo			Interaction *P*-value
Outcome	*n*/*N* (%)	Event rate per 100 person-years	*n*/*N* (%)	Event rate per 100 person-years	Hazard ratio^a^ (95% CI)	
Ischaemic aetiology						
Yes	84/1316 (6.4)	4.4 (3.5–5.4)	113/1358 (8.3)	5.8 (4.8–7.0)	0.76 (0.57–1.00)	0.597
No	56/1057 (5.3)	3.7 (2.8–4.8)	62/1013 (6.1)	4.3 (3.3–5.5)	0.86 (0.60–1.23)	
MRA at baseline						
Yes	110/1696 (6.5)	4.5 (3.7–5.4)	132/1674 (7.9)	5.5 (4.7–6.6)	0.81 (0.63–1.05)	0.621
No	30/677 (4.4)	3.0 (2.1–4.3)	43/697 (6.2)	4.2 (3.1–5.7)	0.71 (0.45–1.14)	
ICD/CRT-D at baseline						
Yes	51/622 (8.2)	5.8 (4.4–7.6)	51/620 (8.2)	5.9 (4.5–7.7)	0.99 (0.67–1.45)	
No	89/1751 (5.1)	3.5 (2.8–4.3)	124/1751 (7.1)	4.9 (4.1–5.8)	0.71 (0.54–0.93)	0.174
Time from diagnosis of HF						
<1 year	19/531 (3.6)	2.5 (1.6–3.8)	30/567 (5.3)	3.7 (2.6–5.3)	0.67 (0.38–1.20)	
≥1year	121/1842 (6.6)	4.5 (3.8–5.4)	145/1804 (8.0)	5.6 (4.8–6.6)	0.81 (0.64–1.03)	0.533
Diabetes mellitus						
Yes	57/993 (5.7)	3.9 (3.0–5.1)	81/990 (8.2)	5.8 (4.7–7.2)	0.69 (0.49–0.96)	0.273
No	83/1380 (6.0)	4.1 (3.3–5.1)	94/1381 (6.8)	4.7 (3.8–5.7)	0.88 (0.66–1.18)	
Previous ventricular arrhythmia						
Yes	26/278 (9.4)	6.7 (4.5–9.8)	34/242 (14.0)	10.1 (7.2–14.1)	0.66 (0.40–1.10)	0.492
No	114/2095 (5.4)	3.7 (3.1–4.5)	141/2129 (6.6)	4.6 (3.9–5.4)	0.81 (0.63–1.04)	
NYHA class						
II	82/1606 (5.1)	3.5 (2.8–4.3)	108/1597 (6.8)	4.7 (3.9–5.7)	0.74 (0.55–0.98)	0.454
III/IV	58/767 (7.6)	5.4 (4.1–6.9)	67/774 (8.7)	6.0 (4.7–7.7)	0.87 (0.61–1.24)	
NT-proBNP (pg/ml)^b^						
≤Median	45/1193 (3.8)	2.5 (1.9–3.4)	76/1179 (6.4)	4.4 (3.5–5.5)	0.58 (0.40–0.84)	0.032
>Median	95/1179 (8.1)	5.7 (4.6–6.9)	99/1191 (8.3)	5.9 (4.9–7.2)	0.96 (0.72–1.27)	
LVEF (%)						
≤Median	87/1230 (7.1)	5.0 (4.0–6.2)	113/1239 (9.1)	6.5 (5.4–7.8)	0.77 (0.58–1.02)	0.740
>Median	53/1143 (4.6)	3.1 (2.4–4.1)	62/1132 (5.5)	3.7 (2.9–4.8)	0.83 (0.58–1.20)	
Systolic BP (mmHg)						
≤Median	87/1171 (7.4)	5.3 (4.3–6.5)	101/1223 (8.3)	5.9 (4.9–7.2)	0.89 (0.67–1.19)	0.226
>Median	53/1202 (4.4)	2.9 (2.2–3.9)	74/1148 (6.4)	4.4 (3.5–5.5)	0.67 (0.47–0.96)	

BP, blood pressure; CI, confidence interval; CRT-D, cardiac resynchronization therapy with defibrillator; HF, heart failure; ICD, implantable cardioverter defibrillator; LVEF, left ventricular ejection fraction; MRA, mineralocorticoid receptor antagonist; NT-proBNP, N-terminal pro-B-type natriuretic peptide; NYHA, New York Heart Association.

a Models included factors for randomized treatment, history of HF hospitalization and were stratified by diabetes status.

b Missing data for *n* = 2 patients.

Of the 2372 patients with a baseline NT-proBNP at or below the median, 121 (5.1%) experienced the primary composite event. A total of 194 of 2370 (8.2%) patients with a baseline NT-proBNP above the median experienced one of the composite events. The HR for the effect of dapagliflozin, compared with placebo, on the composite outcome in patients with an NT-proBNP at or below the median was 0.58 (0.40–0.84) compared with 0.96 (0.72–1.27) in those with an NT-proBNP above the median. The *P*-value for interaction was 0.032 (*Table 4*). When the interaction was tested using NT-proBNP modelled as a continuous variable, the *P*-value for interaction was 0.056.

### Association between change in NT-proBNP and occurrence of any serious ventricular arrhythmia, resuscitated cardiac arrest, or sudden death

Change in NT-proBNP between baseline and 8 months was analysed in 4222 patients (89.0%). An increase in NT-proBNP, adjusted for baseline value, was associated with a higher risk of any serious ventricular arrhythmia, resuscitated cardiac arrest or sudden death [HR per doubling of NT-proBNP, 1.55 (95% CI 1.33–1.81)], whereas a decrease in NT-proBNP was associated with a lower risk [HR per halving of NT-proBNP, 0.65 (95% CI 0.55–0.75)] (Supplementary material online, *Appendix Figure S4*).

### Association between a report of any serious ventricular arrhythmia and subsequent mortality

When the occurrence of a serious ventricular arrhythmia was modelled as a time-varying covariate, there was a strong association with mortality. For a serious ventricular arrhythmia, the unadjusted HR for subsequent death from any causes was 2.16 (95% CI 1.31–3.56) (*P* = 0.002). The corresponding adjusted HR was 2.09 (95% CI 1.26–3.45) (*P* = 0.004).

## Discussion

The main finding of this analysis was that dapagliflozin reduced the risk of the composite outcome of an investigator-reported serious ventricular arrhythmia, resuscitated cardiac arrest, or sudden death in patients with HFrEF (*Graphical Abstract*).

In DAPA-HF, ventricular arrhythmias were identified through adverse event reporting by investigators, rather than by systematic monitoring. Although systematic approaches to arrhythmia detection using techniques such as ambulatory monitoring identify ventricular arrhythmias in most patients with HFrEF, arrhythmias reported spontaneously as SAEs are likely to be the most clinically important. Using a similar analytical approach, we found an almost identical rate of ‘serious’ ventricular arrhythmias in PARADIGM-HF (unpublished data) and the rates of appropriate ICD therapy reported in generally similar patient populations are broadly in keeping with the rates of ‘serious’ ventricular arrhythmias in DAPA-HF and PARADIGM-HF, accounting for background therapy and other differences.^24^ The view that the events reported are more clinically significant is supported by the high subsequent mortality rate in patients with an SAE report of this type in DAPA-HF. We found that an investigator-reported ventricular arrhythmia, analysed as a time-varying covariate, was associated with a doubling of the risk of death and this elevated risk persisted after adjustment for other prognostic variables, including NT-proBNP. The independent predictors of the primary endpoint were NT-proBNP concentration, history of ventricular arrhythmia, LVEF, history of myocardial infarction, body mass index, systolic blood pressure, male sex, and serum sodium concentration, in keeping with prior reports.^24^ ^,^ ^25^

The risk of any ventricular arrhythmia, resuscitated cardiac arrest, or sudden death was decreased by 21% when dapagliflozin was added to other treatments previously shown to reduce ventricular arrhythmias and sudden death, including beta-blockers, MRAs, and sacubitril/valsartan.^25–30^ The findings in patients with and without a defibrillating device (an ICD or CRT-D) were of particular interest. Dapagliflozin appeared to substantially reduce the primary endpoint in patients without a defibrillating device and to have little effect in patients with such devices. However, the *P*-value for the interaction between the effect of dapagliflozin and baseline device status was non-significant and the device subgroup was of modest size and had relatively few events. However, it is plausible that dapagliflozin might be less likely to reduce sudden death in patients with an ICD/CRT-D compared to those without such a device. The effect of dapagliflozin on a ventricular arrhythmia or sudden death seemed to be more consistent regarding aetiology (non-ischaemic vs. ischaemic) and baseline MRA therapy (we did not perform a subgroup analysis for renin-angiotensin system blockers or beta-blockers because >90% of participants were receiving both of these therapies).

We do not know of any other reports of the effect of SGLT2 inhibitors on ventricular arrhythmias in this patient group, although empagliflozin has been shown to reduce the incidence of VF related to ischaemia/reperfusion in Langendorff-perfused rabbit hearts.^31^ There are several potential mechanisms by which SGLT2 inhibitors might reduce ventricular arrhythmias. First, favourable haemodynamic and remodelling actions may have an indirect effect as chamber dilatation and cardiomyocyte stretch are associated with the occurrence of arrhythmias. In three randomized trials in patients with HFrEF, empagliflozin reduced cardiac chamber size and SGLT2 inhibitors also reduce NT-proBNP levels, suggesting that they decrease wall stress, at least in part due to their diuretic action.^11–15^ Consistent with this hypothesis, we found that both LVEF and NT-proBNP were independent predictors of the composite outcome and increasing NT-proBNP over time was associated with a higher risk of ventricular arrhythmias, resuscitated cardiac arrest, or sudden death. That the separation of the Kaplan–Meier curves for this composite outcome was clearest after 9 months might be consistent with a role for favourable cardiac remodelling in the benefit of dapagliflozin. However, the relationship with NT-proBNP is complex in that while higher NT-proBNP levels do predict a greater risk of sudden death, NT-proBNP is a relatively more powerful predictor of death due to progressive pump failure.^32^ Sodium-glucose cotransporter 2 inhibitors may also have a favourable effect on the autonomic nervous system. In the EMBODY trial,^16^ empagliflozin improved both sympathetic and parasympathetic activities in patients with myocardial infarction and type 2 diabetes, a finding supported by a study with dapagliflozin in a pig model of heart failure.^33^ Empagliflozin does not prolong QT interval in healthy humans and attenuated sotalol-induced QTc prolongation in rats.^34^ ^,^ ^35^ Experimentally, SGLT2 inhibitors also inhibit the cardiac sodium channel current (late *I*_Na_) in cardiomyocytes from mice with heart failure and in cardiac Nav1.5 sodium channels containing the long QT syndrome 3 mutations.^18^ More controversially, SGLT2 inhibitors may inhibit the myocardial sodium/hydrogen (Na^+^/H^+^) exchanger-1 (NHE-1), although this has not been confirmed by all investigators.^36–39^ Additional possibilities include a favourable effect on myocardial metabolism and energetics and a reduction in epicardial adipose tissue, which may be associated with a greater risk of arrhythmias.^40–42^

Our study had several limitations. First, this analysis was not prespecified. As a result, our findings should be regarded as hypothesis generating and require confirmation, although a recent meta-analysis of 22 trials involving 52 115 patients with a variety of conditions (including type 2 diabetes, chronic kidney disease, and heart failure) found that the use of an SGLT2 inhibitor was associated with a lower risk of VT reported as an adverse event (relative risk 0.73, 95% CI 0.53–0.99).^43^ Second, adverse event reporting likely underestimated the true prevalence of ventricular arrhythmias. We did not have information on ICD discharges. We were unable to tell whether the higher incidence of ventricular arrhythmias in patients with devices reflected why the device was implanted (i.e. for primary prevention in a high-risk patient or because of a prior arrhythmia) or because these devices detect arrhythmias. Arrhythmias were not adjudicated although a prior similar study showed a reduction in arrhythmias with a beta-blocker, consistent with the effect found in studies using systematic monitoring.^44^ Although ventricular arrhythmias are clearly linked with sudden death, not all sudden deaths are due to an arrhythmia (or any electrical disturbance), which is why the risk of sudden death is not eliminated by ICDs. Conversely, ventricular arrhythmias are also predictive of non-sudden death as they are often a marker of more advanced heart failure.

In summary, in this *post hoc* analysis, dapagliflozin, compared with placebo, reduced the incidence of investigator-reported (but not adjudicated) ventricular arrhythmias in patients with HFrEF, most of whom were treated with a renin–angiotensin system blocker, beta-blocker, and an MRA as well.

## Supplementary material

Supplementary material is available at *European Heart Journal* online.

## Funding

The DAPA-HF trial was funded by AstraZeneca. J.J.V.M. and M.C.P. are supported by a British Heart Foundation Centre of Research Excellence Grant (RE/18/6/34217).

**Conflict of interest:** J.P.C. has no disclosures. K.F.D.’s employer, the University of Glasgow, has been remunerated by AstraZeneca for working on the DAPA-HF trial. P.S.J.’s employer, the University of Glasgow, has been remunerated by AstraZeneca for working on the DAPA-HF trial (Dapagliflozin and Prevention of Adverse-Outcomes in Heart Failure) and the DELIVER trial (Dapagliflozin Evaluation to Improve the Lives of Patients With Preserved Ejection Fraction Heart Failure) and speakers and advisory board fees from AstraZeneca. Speakers and advisory board fees from Novartis and advisory board fees and grants from Boehringer Ingelheim. M.C.P. reports receiving lecture fees from AstraZeneca and Eli Lilly during the conduct of the study and personal fees from Novo Nordisk, AstraZeneca, NAPP Pharmaceuticals, Takeda Pharmaceutical, Alnylam, Bayer, Resverlogix, and Cardiorentis, and grants and personal fees from Boehringer Ingelheim and Novartis. S.E.I. reports personal fees and nonfinancial support from AstraZeneca, Boehringer Ingelheim, Sanofi/Lexicon, Merck, VTV Therapeutics, and Abbott/Alere, and personal fees from AstraZeneca and Zafgen, as well. L.K. reports other support from AstraZeneca and personal fees from Novartis and Bristol Myers Squibb as a speaker. M.N.K. reports personal fees from AstraZeneca and Vifor Pharma; grants, personal fees, and other from AstraZeneca; grants and personal fees from Boehringer Ingelheim; and personal fees from Sanofi, Amgen, Novo Nordisk, Merck (Diabetes and Cardiovascular), Janssen, Bayer, Applied Therapeutics, and Eli Lilly. F.A.M. reports personal fees from AstraZeneca. M.S.S. reports grants from Bayer, Daiichi Sankyo, Eisai, GlaxoSmithKline, Pfizer, Poxel, Quark Pharmaceuticals, and Takeda; grants and personal fees from Amgen, AstraZeneca, Intarcia, Janssen Research and Development, The Medicines Company, MedImmune, Merck, and Novartis; and personal fees from Anthos Therapeutics, Bristol Myers Squibb, CVS Caremark, DalCor, Dyrnamix, Esperion, IFM Therapeutics, and Ionis. P.P. reports personal fees and other from AstraZeneca, Boehringer Ingelheim, Bayer, BMS, Cibiem, Novartis, and RenalGuard; personal fees from Pfizer, Servier, Respicardia, and Berlin-Chemie; other from Amgen; and grants, personal fees, and other from Vifor Pharma. M.S.S. is a member of the TIMI Study Group, which has also received institutional research grant support through Brigham and Women’s Hospital from Abbott, Aralez, Roche, and Zora Biosciences. O.B., A.M.L., and M.S. are employees of AstraZeneca. S.D.S. has received research grants from Actelion, Alnylam, Amgen, AstraZeneca, Bellerophon, Bayer, BMS, Celladon, Cytokinetics, Eidos, Gilead, GSK, Ionis, Lilly, Lone Star Heart, Mesoblast, MyoKardia, NIH/NHLBI, NeuroTronik, Novartis, Novo Nordisk, Respicardia, Sanofi Pasteur, Theracos, and has consulted for Abbott, Action Akros, Alnylam, Amgen, Arena, AstraZeneca, Bayer, Boehringer Ingelheim, BMS, Cardior, Cardurion, Corvia, Cytokinetics, Daiichi Sankyo, Gilead, GSK, Ironwood, Lilly, Merck, Myokardia, Novartis, Roche, Takeda, Theracos, Quantum Genetics, Cardurion, AoBiome, Janssen, Cardiac Dimensions, Tenaya, Sanofi Pasteur, Dinaqor, Tremeau, CellProThera, Moderna, and American Regent. J..J.V.M.’s employer, the University of Glasgow, has been remunerated by AstraZeneca for working on the DAPA-HF and DELIVER trial, Cardiorentis, Amgen, Oxford University/Bayer, Theracos, Abbvie, Novartis, Glaxo Smith Kline, Vifor-Fresenius, Kidney Research UK, and Novartis, and other support from Bayer, DalCor, Pfizer, Merck, Bristol Myers, and Squibb, as well.

### Data availability

The data that support the findings of this study are available from the corresponding author on reasonable request.

## Supplementary Material

ehab560_Supplementary_Data
