# Supplementary material for: Effect of dapagliflozin on ventricular arrhythmias, resuscitated cardiac arrest, or sudden death in DAPA-HF
Source: Eur Heart J. 2021 Aug 27;42(36):3727–38. doi: 10.1093/eurheartj/ehab560 (PMC8455345; doi:10.1093/eurheartj/ehab560)

SUPPLEMENTARY APPENDIX

Table S1. Baseline characteristics of participants who had no ventricular arrhythmia compared with those who had a serious ventricular arrhythmia* and a sudden death

	**No serious ventricular arrhythmia or sudden death**	**Serious ventricular arrhythmia****	**p Value**	**Sudden death*****	**p Value**
**n = (%)#**	4429 (93.4)	115 (2.4)		206 (4.3)	
**Age (years)**	66 ± 11	66 ± 10	0.710	66 ± 11	0.760
**Race (%)**			<0.001		0.600
**White**	3097 (69.9)	90 (78.3)		153 (74.3)	
**Black**	210 (4.7)	6 (5.2)		9 (4.4)	
**Asian**	1060 (23.9)	13 (11.3)		42 (20.4)	
**Other**	62 (1.4)	6 (5.2)		2 (1.0)	
**Region (%)**			0.039		0.012
**North America**	639 (14.4)	21 (18.3)		17 (8.3)	
**South America**	751 (17.0)	21 (18.3)		47 (22.8)	

**Europe**	1999 (45.1)	59 (51.3)		101 (49.0)	
**Asia-Pacific**	1040 (23.5)	14 (12.2)		41 (19.9)	
**Sex (%)**			0.240		0.001
**Male**	3371 (76.1)	93 (80.9)		177 (85.9)	
**SBP (mmHg)**	122 ± 16	116 ± 15	<0.001	119 ± 15	0.012
**Heart Rate (bpm)- Sinus**	71 ± 11	68 ± 12	0.066	73 ± 13	0.730
**Heart Rate (bpm)- AF**	73 ± 13	70 ± 10	0.074	73 ± 11	0.040
**BMI (kg/m^2)**	28 ± 6	29 ± 6	0.280	28 ± 7	0.960
**eGFR (ml/min/1.73m^2)**	66 ± 19	63 ± 16	0.170	62 ± 19	0.005
**eGFR <60 ml/min/1.73m^2**	1780 (40.2)	50 (43.5)	0.480	99 (48.1)	0.025
**LVEF (%) (IQR)**	32 (26 – 37)	29 (22 – 33)	<0.001	30 (25 – 35)	<0.001
**LVEF**			<0.001		0.035
**≤ median**	2269 (51.2)	83 (72.2)		121 (58.7)	
**> median**	2160 (48.8)	32 (27.8)		85 (41.3)	

**NT-proBNP (pg/ml) (IQR)- not AF/F**	1238 (735 – 2259)	1443 (877 – 3056)	0.030	2344 (1068 – 4753)	<0.001
**NT-proBNP (pg/ml) (IQR)- AF/F**	1777 (1099 – 2998)	1497 (1088 – 2770)	0.280	2864 (1348 – 5136)	<0.001
**Potassium (mmol/L)**	4.5 ± 0.5	4.5 ± 0.5	0.220	4.5 ± 0.6	0.490
**Sodium (mmol/L)**	140 ± 3	139 ± 3	0.028	139 ± 3	0.003
**QRS duration (ms)**	121 ± 36	129 ± 31	0.030	125 ± 33	0.150
**QRS duration ≥ 130 ms**	1508 (34.0)	49 (42.6)	0.056	83 (40.3)	0.065
**QRS duration ≥ 150 ms**	964 (21.8)	28 (24.3)	0.510	56 (27.2)	0.066
**NYHA Class (%)**			0.540		<0.001
**II**	3013 (68.0)	79 (68.7)		116 (56.3)	
**III**	1380 (31.2)	34 (29.6)		86 (41.7)	
**IV**	36 (0.8)	2 (1.7)		4 (1.9)	

**KCCQ-TSS (IQR)**	78 (59 – 92)	77 (60 – 90)	0.630	71 (54 – 88)	<0.001
**Duration of heart failure**			<0.001		0.130
**<1 year**	1049 (23.7)	10 (8.7)		41 (19.9)	
**1 – 5 years**	1681 (38.0)	37 (32.2)		72 (35.0)	
**>5 years**	1699 (38.4)	68 (59.1)		93 (45.1)	
**Ischaemic aetiology**	2477 (55.9)	65 (56.5)	0.900	133 (64.6)	0.015
**Medical history (%)**					
**Previous ventricular arrhythmia**	460 (10.4)	35 (30.4)	<0.001	28 (13.6)	0.140
**Hypertension**	3291 (74.3)	79 (68.7)	0.170	156 (75.7)	0.650
**Diabetes mellitus**	1845 (41.7)	38 (33.0)	0.064	99 (48.1)	0.069
**AF history**	1693 (38.2)	49 (42.6)	0.340	78 (37.9)	0.920
**AF / Flutter on baseline ECG**	1050 (23.7)	24 (20.9)	0.480	55 (26.7)	0.320

**Prior HF hospitalization**	2100 (47.4)	58 (50.4)	0.520	94 (45.6)	0.620
**MI**	1926 (43.5)	58 (50.4)	0.140	111 (53.9)	0.003
**PCI**	1498 (33.8)	51 (44.3)	0.019	75 (36.4)	0.440
**CABG**	736 (16.6)	24 (20.9)	0.230	42 (20.4)	0.160
**Stroke**	426 (9.6)	13 (11.3)	0.550	28 (13.6)	0.061
**COPD**	544 (12.3)	12 (10.4)	0.550	29 (14.1)	0.440
**Anaemia†**	1208 (27.3)	29 (25.2)	0.620	67 (32.5)	0.099
**CV therapy (%)**					
**Loop diuretic**	3549 (80.1)	100 (87.0)	0.069	180 (87.4)	0.010
**Thiazide diuretic**	443 (10.0)	9 (7.8)	0.440	24 (11.7)	0.440
**ARNI**	465 (10.5)	27 (23.5)	<0.001	16 (7.8)	0.210
**ACE inhibitor**	2489 (56.2)	50 (43.5)	0.007	127 (61.7)	0.120
**ARB**	1229 (27.7)	33 (28.7)	0.820	46 (22.3)	0.089
**Beta-blocker**	4263 (96.3)	112 (97.4)	0.520	190 (92.2)	0.004

**MRA**	3128 (70.6)	90 (78.3)	0.075	158 (76.7)	0.061
**Digoxin**	827 (18.7)	16 (13.9)	0.190	45 (21.8)	0.250
**Amiodarone**	135 (3.0)	6 (5.2)	0.190	10 (4.9)	0.150
**Sotalol**	9 (0.2)	2 (1.7)	<0.001	0 (0.0)	0.520
**ICD**	1140 (25.7)	68 (59.1)	<0.001	38 (18.4)	0.019
**CRT- D**	282 (6.4)	12 (10.4)	0.080	1 (0.5)	0.360
**Diabetes therapy (%)**††					
**Biguanide**	953 (51.7)	16 (42.1)	0.049	48 (48.5)	0.540
**Sulphonylurea**	411 (22.3)	4 (10.5)	0.033	23 (23.2)	0.360
**DPP4 inhibitor**	284 (15.4)	7 (18.4)	0.890	18 (18.2)	0.190
**GLP-1 agonist**	20 (1.1)	0 (0.0)	0.470	1 (1.0)	0.940
**Insulin**	503 (27.3)	9 (23.7)	0.24	29 (29.3)	0.230

*Serious ventricular arrhythmia was defined as any serious adverse event report using the MedDRA preferred terms “ventricular tachycardia”, “ventricular fibrillation”, “torsade de pointes”, “ventricular tachyarrhythmia” and “ventricular arrhythmia”. Premature ventricular ectopics were excluded.

**Excluded patients who had a sudden death or resuscitated cardiac arrest and did not have a serious ventricular arrhythmia (n=200)

***Excluded patients who had a serious ventricular arrhythmia or resuscitated cardiac arrest and did not have a sudden death (n=109)

#Calculated % value out of total study cohort, n=4744

|| Plus-minus values are means ± standard deviations. IQR denotes interquartile range

†Anaemia was defined as Hb <130 g/L in males and Hb <120 g/L in females

†† Per cent is of patients with a history of diabetes

ACE = angiotensin converting enzyme; AF/F = atrial fibrillation/atrial flutter; ARB = angiotensin receptor blocker; ARNI = angiotensin receptor blocker neprilysin inhibitor; BMI = body mass index; CABG = coronary bypass graft; COPD = chronic obstructive pulmonary disease; CRT-D = cardiac resynchronization therapy defibrillator; CRT-P = cardiac resynchronization therapy pacemaker; DPP4 = dipeptidyl peptidase-4; ECG = electrocardiogram; eGFR = estimated glomerular filtration rate; GLP-1 = glucagon-like peptide 1; HF = heart failure; ICD = implantable cardioverter defibrillator; KCCQ CSS = Kansas City Cardiomyopathy Questionnaire clinical summary score; LVEF = left ventricular ejection fraction; MI = myocardial infarction; MRA = mineralocorticoid receptor antagonist; NT-proBNP = N-terminal pro B-type natriuretic peptide; NYHA = New York Heart Association; PCI = percutaneous coronary intervention; SBP = systolic blood pressure

Table S2. Competing risks regression for time-to-first serious ventricular arrhythmia / resuscitated cardiac arrest / sudden death with all-cause death as a competing risk (Fine and Gray model)

	**Dapagliflozin**	**Placebo**	
**Outcome**	**n/N(%)**	**n/N(%)**	**Sub-Hazard Ratio***
**Serious ventricular arrhythmia / resuscitated cardiac arrest / sudden death**	140/2373 (5.9)	175/2371 (7.4)	0.80 (0.64-0.99); p=0.043
Serious ventricular arrhythmia	50/2373 (2.1)	65/2371 (2.7)	0.77 (0.53-1.11)
Resuscitated cardiac arrest	5/2373 (0.2)	3/2371 (0.1)	-
Sudden death	93/2373 (3.9)	113/2371 (4.8)	0.82 (0.62-1.08)
**VT / VF / torsade de pointes / resuscitated cardiac arrest or sudden death**	134/2373 (5.6)	171/2373 (7.2)	0.78 (0.62-0.98); p=0.030
VT / VF / torsade de pointes	60/2373 (2.5)	44/2371 (1.9)	0.73 (0.50-1.08)

***Models** included factors for randomized treatment, history of heart failure hospitalization and were stratified by diabetes status; a hazard ratio was not calculated because there were fewer than 10 events overall.

Figure S1. Relationship between baseline NT-proBNP, modelled as a continuous variable, and the risk of any serious ventricular arrhythmia, resuscitated cardiac arrest or sudden death. The referent value is the study cohort median NT-proBNP value (approximately1440 pg/ml)

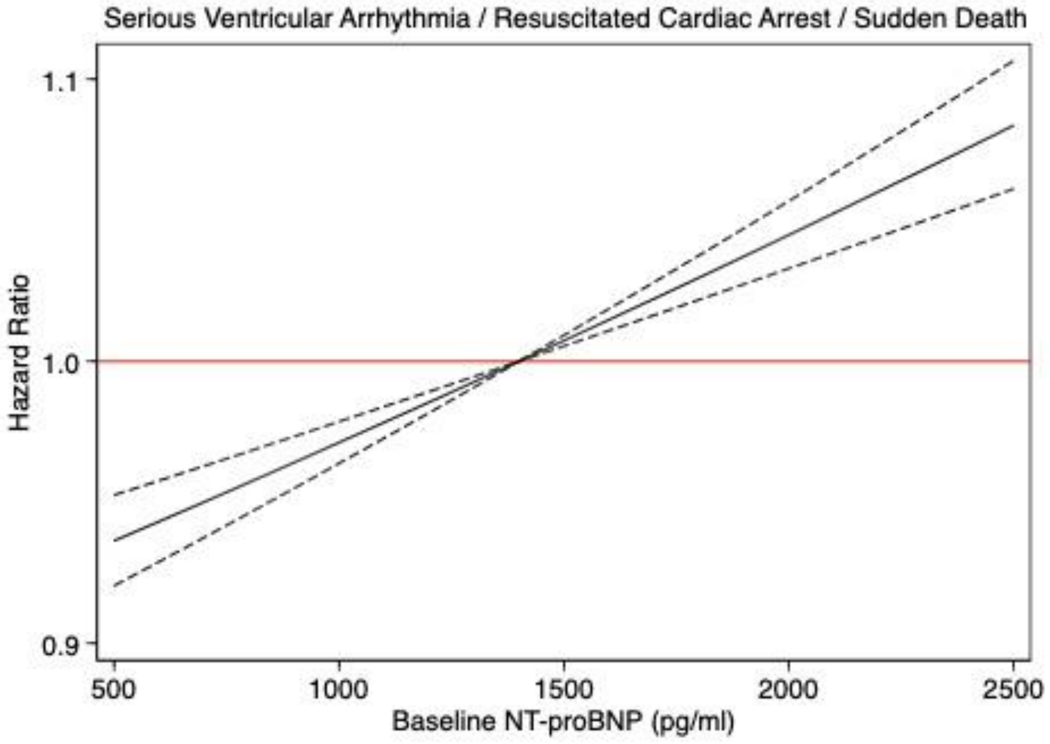

Figure S2. Relationship between baseline left ventricular ejection fraction (LVEF), modelled as a continuous variable, and the risk of any serious ventricular arrhythmia, resuscitated cardiac arrest or sudden death. The referent value is the study cohort median LVEF (32%).

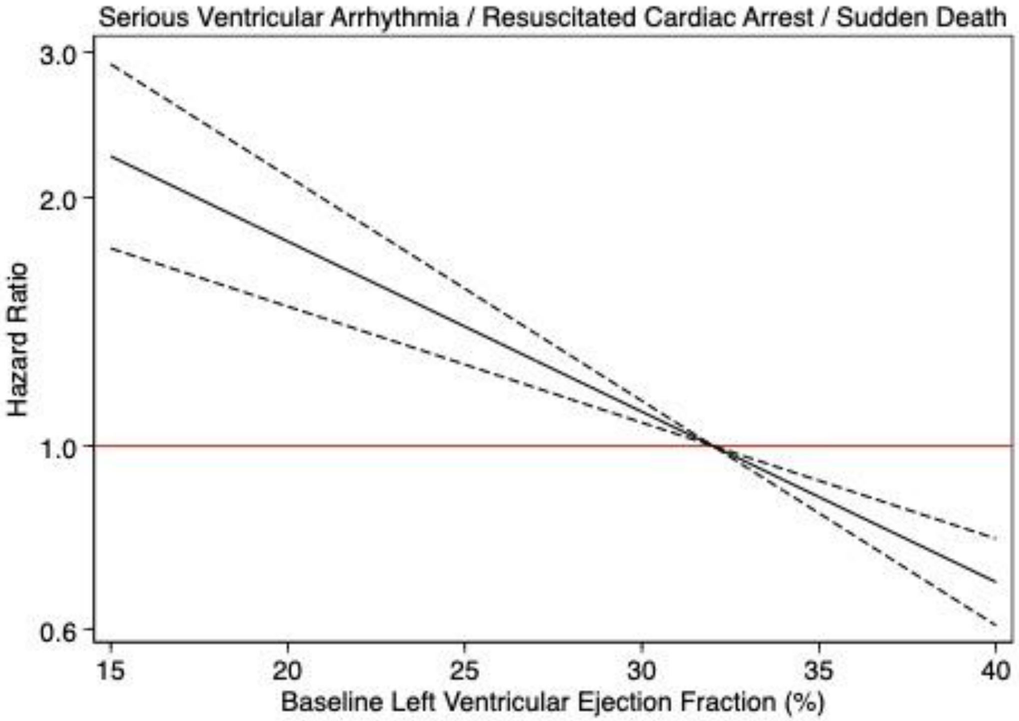

Figure S3. Cumulative incidence of a first serious ventricular arrhythmia, resuscitated cardiac arrest or sudden death in a competing risks regression according to treatment assignment.

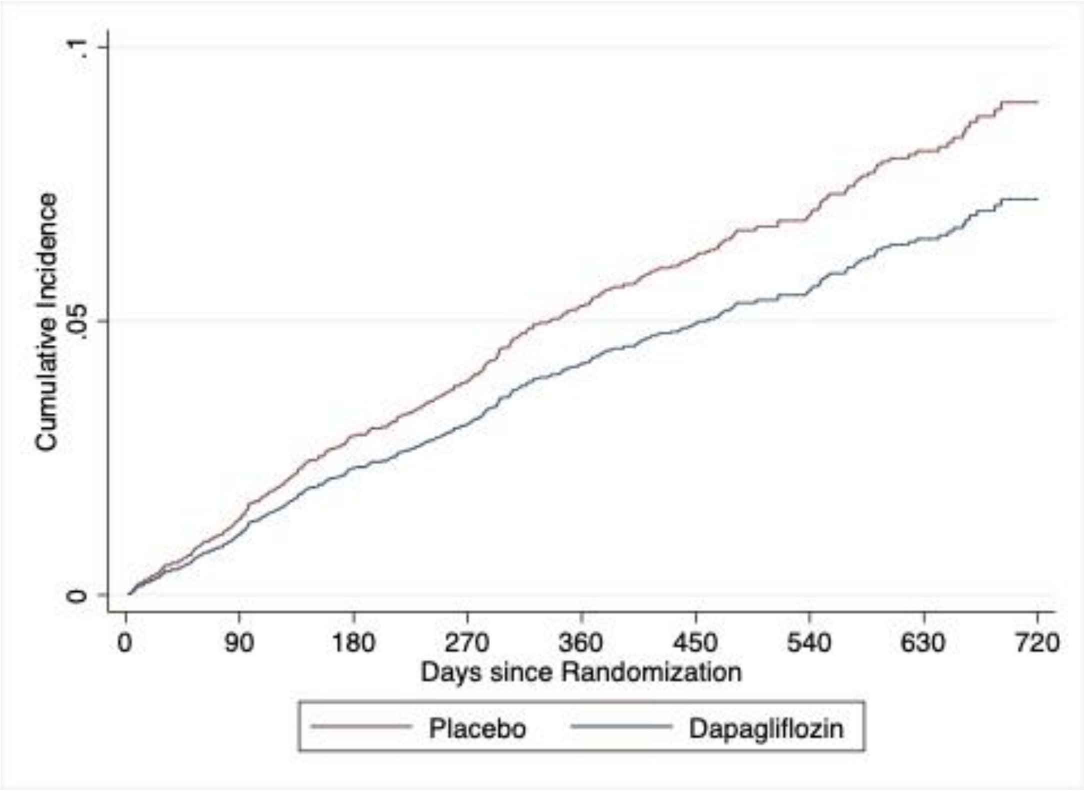

Figure S4. Linear model of the relationship between relative changes in log_2-transformed NT-proBNP and the incidence of any serious ventricular arrhythmia, resuscitated cardiac arrest or sudden death**.** Changes from baseline data are presented as Log2(NT-proBNP at 8 months after randomization / baseline NT-proBNP). A 0 value represents no change from baseline, +1 represents a doubling of NT-proBNP at 8 months compared with baseline, and -1 represents a halving of NT-proBNP at 8 months compared with baseline.

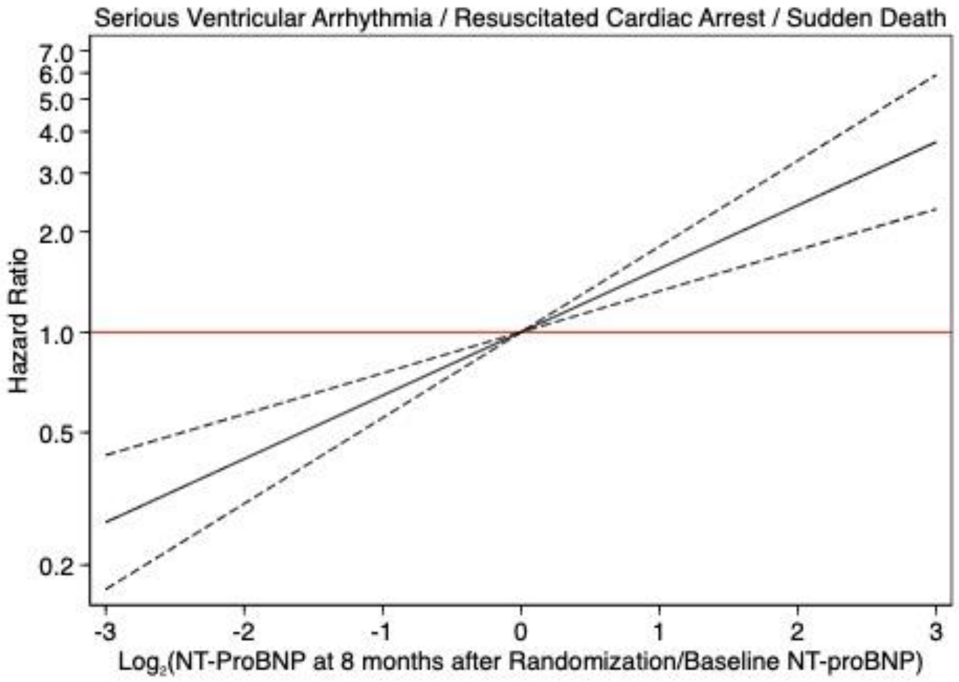

Supplement: ehab560_Supplementary_Data [file ehab560_supplementary_data.pdf]
